# Special Issue “Diabetes: Comorbidities, Therapeutics and Insights”

**DOI:** 10.3390/biomedicines13092043

**Published:** 2025-08-22

**Authors:** Tomislav Bulum

**Affiliations:** 1 Department of Diabetes and Endocrinology, Vuk Vrhovac University Clinic for Diabetes, Endocrinology and Metabolic Diseases, Merkur University Hospital, 10000 Zagreb, Croatia; tomobulum@gmail.com; Tel.: +385-12353887; Fax: +385-12331515; 2 School of Medicine, University of Zagreb, Šalata 3, 10000 Zagreb, Croatia

Despite increasing awareness of diabetes and its devastating complications, it remains one of the leading causes of death and disability worldwide, irrespective of gender, age, and country [1]. It is estimated that in 2021, about 537 million people across the world suffered from diabetes, and in the following decades, a further increase to 783 million people suffering from diabetes by 2045 is assumed [2]. In addition, health expenditures associated with diabetes, primarily connected with the treatment of chronic complications of diabetes, will reach more than USD 1054 billion by 2045 [3].

The most devastating effects of diabetes represent its chronic complications, generally divided into microvascular complications (diabetic kidney disease, diabetic retinopathy, and diabetic neuropathy) and macrovascular complications (cardiovascular diseases) [4]. Diabetes is still the most common cause of blindness among the adult working population [5]. Diabetes is the leading cause of kidney disease, dialysis, and kidney transplantation [6]. About one out of three adults with diabetes has kidney disease. Diabetes is the leading cause of nontraumatic leg amputations, and more than 60% of non-traumatic lower limb amputations happen in patients with diabetes [7]. Diabetic foot ulcers significantly increase the risk of limb amputations and are associated with micro- and macrovascular dysfunction [8].

While microvascular complications of diabetes are the most important risk factors for disability, macrovascular diseases (myocardial infarction, stroke, and peripheral arterial disease) are the most important risk factors for premature morbidity and mortality [9]. Patients with diabetes have a two- to four-fold excess risk of heart disease or stroke compared to people without diabetes and are more vulnerable to premature death [10]. The high risk of cardiovascular disease and death in people with diabetes is a consequence of several risk factors present in people with diabetes and connected with obesity, like hypertension, hyperlipidemia, and hyperglycemia. All these risk factors are usually present in patients with type 2 diabetes and are connected with a high risk of cardiovascular morbidity and mortality [10]. Nowadays, the term Cardiovascular–Kidney–Metabolic (CKM) syndrome by the American Heart Association (AHA) has been introduced to emphasize interconnections between cardio-renal–metabolic diseases [11]. It is essential to note that, besides optimal glucose control, strict control of other risk factors like hypertension, hyperlipidemia, obesity, smoking, and other cardiovascular risk factors is needed to reduce the risk of cardiovascular disease and premature death [12].

Sodium-glucose cotransporter 2 (SGLT-2) inhibitors and glucagon-like peptide-1 receptor (GLP-1) agonists are a new class of antidiabetic drugs that are encouraged to use by cardiology, nephrology, and diabetology guidelines because of their favorable cardiorenoprotective effects, including a decreased risk of chronic kidney disease progression [13]. However, patients treated with GLP-1 agonists have a higher rate of progression of diabetic retinopathy, so those patients must be closely monitored for retinopathy status to decrease the risk of vision-threatening complications [14,15].

Generally, although there has been a trend of declines in several diabetes complications recently, like all-cause and cardiovascular-related mortality, those suffering from diabetes still have a significantly higher risk for these complications compared with those without diabetes [4]. Considering all these, additional efforts are needed to reduce the risk of comorbidities and premature death in patients with diabetes, and this Special Issue aims to present the latest research and advances in diabetes comorbidities, therapeutics, and insights comprising thirteen research articles, three review articles, and one systematic review.

The first research article focuses on one of the current hot-topic drugs in the treatment of diabetes and obesity, semaglutide. Social media significantly increases the risk of the off-label use of semaglutide for obesity. They found that semaglutide, compared to other GLP-1 agonists, had the highest proportions of searches on Google associated with the term “weight loss” and had the lowest number of severe adverse drug reactions, but it also had the highest number of individual case safety reports reported in the EudraVigilance database. Because of a high tendency for overdose reports with semaglutide, there is a high risk of improper or off-label use of semaglutide that should be reduced and carefully monitored.

The second research article was a retrospective cohort study that investigated depressive symptoms associated with peripheral artery disease and predicting mortality in type 2 diabetes. The study included a large cohort of subjects aged over 60 years, divided according to the five-item Geriatric Depression Scale, developed and validated for screening depressive symptoms, and ankle–brachial index as a measure of peripheral arterial disease. The results of the study suggest that the prevalence of peripheral arterial disease was increased in patients with depressive symptoms, and vice versa. Patients with depression and peripheral arterial disease had the highest risk of long-term mortality.

The third research article investigated the relationship between 44 serum cytokines and growth factors in patients with type 1 diabetes, using the time in ranges and glucose variability, continuous glucose monitoring gold standard parameters for glucoregulation. The changes in the levels of cytokines and growth factors were related to the time-dependent and amplitude-dependent characteristics of glucose variability, suggesting that the pro-inflammatory effect of glucose variability and high glycemia might be connected with changes in cytokine and chemokine production.

The fourth research article explores the correlation between the homeostasis model assessment of insulin resistance (HOMA-IR), as a measure of islet β cell function, and gallbladder stones in patients with newly diagnosed type 2 diabetes mellitus. The results of the study suggest that in both males and females with newly diagnosed type 2 diabetes mellitus, insulin resistance measured with the HOMA-IR index is an independent risk factor for the incidence of gallbladder stones. It is well known that patients with diabetes have a three times higher incidence of gallbladder stones compared to nondiabetics [16]. Researchers deeply investigated that connection and established an accurate predictive model of gallbladder stones in patients with type 2 diabetes that can be an effective tool for the early prediction of gallbladder stones in that population.

The fifth research article explores a significant topic with insufficient research today that connects diabetic kidney disease progression and diabetic gastroenteropathy in patients with long-standing type 1 diabetes. Researchers found that patients with progressive diabetic kidney disease had higher gastrointestinal symptom scores, a newly developed score with 17 questions related to gastrointestinal symptoms, compared to those with stable kidney function. In addition, gastrointestinal symptom scores correlated well with parameters of kidney function, namely estimated glomerular filtration rate and albuminuria. The results of the study suggest that in patients with long-standing type 1 diabetes and progressive diabetic kidney disease, gastrointestinal symptoms worsen in parallel.

The sixth research article examined the relationship between three autoantibodies and their combinations, with anthropometric and metabolic factors, and microvascular complications in patients with latent autoimmune diabetes in adults (LADA). In the present day, patients with LADA are diagnosed much more frequently due to the availability of advanced diagnostic methods and increased awareness of this form of diabetes [17,18]. The results of the study suggest that patients with single autoantibody positivity showed type 2 diabetes characteristics, whereas those positive for two or all three autoantibodies exhibited clinical and metabolic characteristics of type 1 diabetes. The study did not find an association between antibody positivity and microvascular complications of diabetes. In our everyday clinical practice, distinguishing between these two phenotypes in the adult patient population is crucial, as the prevalence of LADA is rising significantly due to increased awareness of this diabetes subtype.

The seventh research article aimed to identify aging-related biomarkers and immune infiltration features in diabetic nephropathy using bioinformatics analysis. Researchers identified a total of 57 differentially expressed aging-related genes that play a major role in inflammatory processes and immunomodulation in diabetic nephropathy. Further analysis identified four differentially expressed aging-related genes (CCR2, VCAM1, CSF1R, and ITGAM) that are involved in immune cell abnormalities and regulate a wide range of immunological and inflammatory responses associated with diabetic nephropathy. Targeting those four potential aging-related genes may be the basis for future therapeutic approaches in diabetic nephropathy.

The eighth research article was a two-year observational study investigating the impact of new antidiabetic drugs, GLP-1 agonists, and dipeptidyl-peptidase-4 inhibitor therapy on hospitalization and mortality in the COVID-19 era. A total of 2910 patients with type 2 diabetes in the Emilia Romagna region required hospitalization (3.8% of the cohort), and 66% of hospitalizations were attributed to COVID-19. Those treated with GLP-1 agonists had lower mortality rates over a two-year observation period compared to any other subgroup treated with various antidiabetic medications, confirming its anti-inflammatory effects regardless of its glucose-lowering effects.

The ninth research article aimed to investigate the effects of deuterium-depleted water on the expression of GLUT4 and glucose uptake, and insulin resistance. Deuterium-depleted water is usually used with chemotherapy and is beneficial in inhibiting cancer development. In patients with diabetes, consumption of deuterium-depleted water has beneficial effects on glucose and insulin levels and HDL cholesterol [19]. This study included cell culture of the mouse myoblast cell line C2C12 and found that deuterium-depleted water promoted the expression and activation of GLUT4 in muscle cells. Consequently, cellular glucose uptake via GLUT4 was maximized, resulting in lower glucose levels and improved insulin sensitivity.

The tenth research article was a retrospective study that investigated neonatal outcomes in patients with gestational diabetes mellitus treated with metformin. Although metformin is usually used in the treatment of gestational diabetes mellitus, there is still skepticism about its effects on fetal outcomes during pregnancy. The study included 234 women with gestational diabetes mellitus and treated with diet, metformin, or insulin. Compared to insulin, metformin was found to be a safe therapy for gestational diabetes mellitus in terms of the rates of neonatal outcomes. Compared to diet, treatment with metformin was associated with a higher risk of neonatal hypoglycemia, neonatal hyperbilirubinemia, large for gestational age, respiratory distress, and the risk of a lower APGAR score. However, after multiple regression analysis, these differences were not statistically significant.

The eleventh research article explores the risk of depression in patients with diabetes among participants in the National Health and Nutrition Examination Survey (NHANES) from 2005 to 2020. It is well known that the risk of depression is increased in patients with diabetes, and the risk might be increased even in those with prediabetes compared to those with normoglycemia [20]. In this study, researchers used the Patient Health Questionnaire-9 (PHQ-9) and HbA1c level from 37,037 individuals. In those with prediabetes, the risk of depression was similar to those with normoglycemia. Patients with diabetes and satisfactory glucose regulation (HbA1c < 5.7%) had a higher risk of mild depression, while those with HbA1c ≥ 10.0% had a higher risk of moderate to severe depression. The results emphasize the need for a holistic approach in the treatment of patients with diabetes, including mental health, particularly for those with HbA1c over 10%.

The twelfth research article focuses on a critical and relevant field in diabetology due to the overall mortality in patients with diabetic foot ulcers being high, with nearly 50% mortality within 5 years [21]. This study aimed to foster a greater understanding of the needs of healthcare professionals treating diabetic foot ulcers using an open-ended questionnaire. While most respondents followed guidelines in the treatment of diabetic foot ulcers, treatment often required a case-specific and holistic approach. This study also emphasizes the complexity of the care of patients with diabetic foot ulcers, suggesting that no single product can address all treatment needs and that products that offer long-lasting antibacterial properties must be cost-effective and easy to use. The education of patients and healthcare professionals is critical for optimal treatment results in patients with diabetic foot ulcers.

The thirteenth research article investigated the connection between Isthmin-1 (Ism1), a novel marker of metabolic health, with metabolic-associated fatty liver disease (MAFLD), insulin resistance, and risk of hyperglycemia in 450 obese and non-obese individuals. Ism1 is an adipokine that plays a role in regulating glucose uptake. The results of this study suggest that reduced Ism1 levels relate to increased insulin resistance, MAFLD, and type 2 diabetes in obese individuals. Low levels of Ism1 are associated with an increased risk of developing prediabetes and type 2 diabetes. Higher Ism1 in obese individuals might be protective against the development of diabetes and other obesity-related metabolic diseases.

The first review deeply investigates the link between stress and the corticotropin-releasing hormone (CRH) system, norepinephrine, depression, and type 2 diabetes. It is well known that major depressive disorder increases the risk of type 2 diabetes by 60%. This review hypothesized that the diminished function of CRH receptor 1 accelerates the CRH–norepinephrine–CRH circuit, which also activates the hypothalamic–pituitary–adrenal axis, leading to increased secretion of norepinephrine and cortisol, and contributing to metabolic and depressive disorders. In addition, the diminished function of CRH receptor 2 could contribute to the pathogenesis of type 2 diabetes and major depressive disorder.

The second review focuses on the role of oxidative stress in tuberculosis meningitis infection in patients with diabetes. Oxidative stress is involved in the pathogenesis of tuberculosis meningitis as well as in the pathogenesis of diabetes and its complications. The use of antioxidants, such as metformin, and other therapies, including vitamin D or glutathione, is a strategy that may improve outcomes in patients with diabetes and prevent the development of tuberculosis meningitis.

The third review explores recent advancements in the management of patients with type 2 diabetes, their place in the new guidelines, and their role in the reduction of both microvascular and macrovascular complications [22]. This review focuses on novel pharmacological therapies, SGLT-2 inhibitors, GLP-1 agonists, and particularly tirzepatide, a new and only dual glucose-dependent insulinotropic polypeptide (GIP) and GLP-1 agonist. Although lifestyle modifications remain foundational in the management of type 2 diabetes, novel pharmacological therapies must be implemented to reduce cardiovascular mortality, invalidity, and prolong life expectancy.

Finally, a network meta-analysis of randomized controlled trials compares the efficacy and tolerability of once-weekly GIP and GLP-1 agonists (semaglutide, exenatide, tirzepatide, and dulaglutide) with once-weekly insulin analogs in the treatment of patients with type 2 diabetes. This analysis included data from 25 randomized controlled trials and 18,257 patients with type 2 diabetes. Tirzepatide was superior to other therapies in reducing HbA1c and weight loss. While GLP-1/GIP agonists were superior in terms of glycemic control and weight reduction, weekly insulins were better tolerated with weight stability.

In conclusion, this Special Issue provides a comprehensive overview of recent developments and research on diabetes, its comorbidities, and therapeutics aimed at a better understanding of and improvement in the treatment of diabetes and its complications. This new knowledge will facilitate the diagnosis of these conditions and may be the basis for future therapeutic approaches.
